# Association of cannabis use with hospital admission and antipsychotic treatment failure in first episode psychosis: an observational study

**DOI:** 10.1136/bmjopen-2015-009888

**Published:** 2016-01-29

**Authors:** Rashmi Patel, Robin Wilson, Richard Jackson, Michael Ball, Hitesh Shetty, Matthew Broadbent, Robert Stewart, Philip McGuire, Sagnik Bhattacharyya

**Affiliations:** 1 Department of Psychosis Studies, King's College London, Institute of Psychiatry, Psychology & Neuroscience, London, UK; 2 Department of Psychological Medicine, King's College London, Institute of Psychiatry, Psychology & Neuroscience, London, UK; 3 South London and Maudsley NHS Foundation Trust, Biomedical Research Centre Nucleus, London, UK

**Keywords:** Natural Language Processing, NLP, Cannabis, CRIS, FEP, Mediation analysis

## Abstract

**Objective:**

To investigate whether cannabis use is associated with increased risk of relapse, as indexed by number of hospital admissions, and whether antipsychotic treatment failure, as indexed by number of unique antipsychotics prescribed, may mediate this effect in a large data set of patients with first episode psychosis (FEP).

**Design:**

Observational study with exploratory mediation analysis.

**Setting:**

Anonymised electronic mental health record data from the South London and Maudsley NHS Foundation Trust.

**Participants:**

2026 people presenting to early intervention services with FEP.

**Exposure:**

Cannabis use at presentation, identified using natural language processing.

**Main outcome measures:**

admission to psychiatric hospital and clozapine prescription up to 5 years following presentation.

**Mediator:**

Number of unique antipsychotics prescribed.

**Results:**

Cannabis use was present in 46.3% of the sample at first presentation and was particularly common in patients who were 16–25, male and single. It was associated with increased frequency of hospital admission (incidence rate ratio 1.50, 95% CI 1.25 to 1.80), increased likelihood of compulsory admission (OR 1.55, 1.16 to 2.08) and greater number of days spent in hospital (β coefficient 35.1 days, 12.1 to 58.1). The number of unique antipsychotics prescribed, mediated increased frequency of hospital admission (natural indirect effect 1.09, 95% CI 1.01 to 1.18; total effect 1.50, 1.21 to 1.87), increased likelihood of compulsory admission (natural indirect effect (NIE) 1.27, 1.03 to 1.58; total effect (TE) 1.76, 0.81 to 3.84) and greater number of days spent in hospital (NIE 17.9, 2.4 to 33.4; TE 34.8, 11.6 to 58.1).

**Conclusions:**

Cannabis use in patients with FEP was associated with an increased likelihood of hospital admission. This was linked to the prescription of several different antipsychotic drugs, indicating clinical judgement of antipsychotic treatment failure. Together, this suggests that cannabis use might be associated with worse clinical outcomes in psychosis by contributing towards failure of antipsychotic treatment.

**Strengths and limitations of this study**

- This is the largest known study (over 2000 participants) to investigate the association of cannabis use with clinical outcome in people with first episode psychosis. As well as demonstrating that cannabis is associated with substantially worse clinical outcomes, our study is the first to identify a possible explanation for these findings through a failure of antipsychotic treatment.
- Our study employed a novel text mining method to identify cannabis use in routinely recorded electronic health record. This approach benefits from increased generalisability of our findings to everyday clinical practice but is limited by the fact that the presence or absence of cannabis use may not have been comprehensively documented in all patients. This may have led to underestimation of its use.
- It was not possible to obtain data on amount, frequency and discontinuation of cannabis use following first presentation to mental health services. Despite this limitation, our data still showed a significant association of cannabis use at presentation to mental health services with poor clinical outcomes up to 5 years later.
- We performed an exploratory mediation analysis to investigate whether the association of cannabis use with poor clinical outcomes could be mediated by an increase in the number of unique antipsychotics prescribed (a marker of antipsychotic treatment failure). However, as this was an observational study, the mediation analysis may have been biased by unmeasured confounders and temporal ambiguity between the mediator and outcome variable.

## Introduction

Cannabis remains the third most common drug of dependence in the world after tobacco and alcohol,^1^ with a growing consensus that cannabis use is associated with increased risk of development of psychotic illnesses particularly if used in early adolescence.^2^ ^3^ However, there is much less agreement regarding its effect on outcome in those with established psychosis, a substantial proportion of whom use the drug, especially in the early stages of psychosis.^4^ Despite the widely held view among clinicians that comorbid cannabis use is a predictor of poor outcome in those with psychosis, the evidence to date has been inconsistent irrespective of the specific outcome measure examined, such as severity of psychotic symptoms or relapse of illness (as indexed by change in symptom severity or hospitalisation), perhaps limited to a large extent by the size of samples and duration of follow-up.^5–10^ Since use of cannabis is potentially amenable to treatment, there is a particular need to definitively investigate the effect of comorbid cannabis use on a robust measure of outcome which is indicative of relapse, such as hospitalisation. This is a reliably estimated measure, and has significant implications for the utilisation of healthcare resources.^11^

Furthermore, questions remain as to how cannabis use may increase the risk of relapse. While increased severity of symptoms is likely to play a role, other (but not necessarily unrelated) mechanisms may be through an adverse effect on adherence,^5^ ^12^ as well as reduced response to antipsychotic treatment.^13^ In a naturalistic setting where decisions regarding medication change take into account a number of factors including response to treatment as well as tolerability and side effects,^14^ the number of unique antipsychotics prescribed is a proxy measure which may encompass all these factors. Hence, compared with someone prescribed fewer unique antipsychotics, a person prescribed a greater number of unique antipsychotics may be considered to have a worse antipsychotic response, or in effect, antipsychotic treatment failure, as a result of either treatment resistance or tolerability to the antipsychotic, or a combination of both. However, whether the effect of cannabis use on the increased risk of relapse in psychosis is partly mediated by its effect on antipsychotic treatment failure (as indexed by the number of unique antipsychotics prescribed) has yet to be investigated. Understanding how cannabis use may adversely affect outcome in psychosis is particularly important as it may identify mechanisms that may potentially be amenable to intervention.

In the present study, we attempt to address these issues by investigating the prevalence of cannabis use and its effect on a cohort of patients with first episode psychosis (FEP) receiving mental healthcare from early intervention services. We employed novel data mining and natural language processing (NLP) tools that allowed us to investigate a large data set of anonymised free-text electronic health records in order to obtain data on cannabis use and clinical outcomes. We tested our hypotheses that in those presenting with their first episode of psychosis, cannabis use is associated with increased frequency of hospital admission (including compulsory admission) and greater number of days spent in hospital, and that this is mediated by non-responsiveness to antipsychotics as indexed by the number of unique antipsychotic medications prescribed.

## Methods

### Participants

All individuals with FEP who were accepted by an early intervention service in the South London and Maudsley (SLaM) National Health Service (NHS) Foundation Trust between 1 April 2006 and 31 March 2013 were included in the study (n=2026). SLaM is one of the largest providers of specialist mental healthcare in Europe, serving a catchment of around 1.2 million residents in four boroughs of South London (Lambeth, Southwark, Lewisham and Croydon).^15^ ^16^

### Source of clinical data

Data for this study were obtained from the SLaM Biomedical Research Centre (BRC) Case Register, which contains anonymised clinical data from the electronic health records of individuals who have previously received or are currently receiving mental healthcare from SLaM.^15^ The SLaM BRC Case Register comprises structured fields for demographic information as well as unstructured (but de-identified) free-text fields from case notes and correspondence where history, mental state examination, diagnostic formulation and management plan are primarily recorded. A patient-led oversight committee considers all proposed research before access to the anonymised data is permitted. The electronic health record system was implemented in SLaM early intervention services in April 2006, and so the period of 1 April 2006 to 31 March 2013 was chosen for data capture to maximise the number of participants with at least 1 year of follow-up. Predictor, covariate and outcome variable data were obtained from the SLaM BRC Case Register using the Clinical Record Interactive Search tool (CRIS),^15^ a search and database assembly tool underpinning this data resource.^17–20^

### Identification of cannabis use

NLP was used to extract documentation of cannabis use from unstructured free-text fields in the BRC Case Register including clinical assessments, reviews and correspondence between healthcare professionals. An NLP application was developed using TextHunter software.^21^ Full details of NLP application development are described in a previous study.^22^ In summary, a support vector machine learning (SVM) approach was used to identify sentences containing a positive reference of current or historical cannabis use. The application was trained using 478 human-classified sentences which contained the word ‘cannabis’ (or the following synonyms: ‘marijuana’, ‘weed’, ‘pot’, ‘hash’, ‘skunk’, ‘resin’) and optimised using two rounds of active learning classification of a further 1357 sentences. The resulting application was tested against a reference standard of 233 human-classified sentences and an SVM marginal filter applied to obtain a minimum precision value (equivalent to positive predictive value) of 90%. As frequency and amount of cannabis use was not documented in electronic health records in the BRC Case Register, a binary variable defined as any documentation of cannabis use by the patient at presentation with FEP was used. In order to establish baseline cannabis use at the time of presenting with FEP, the cannabis NLP application was applied to clinical records documented within 1 month of presentation to early intervention service as, by this time, all patients would have completed a detailed clinical assessment including assessment of substance use history, allowing a reliable estimation of cannabis exposure at presentation with FEP.

### Clinical outcome measures and covariates

The primary outcome was number of psychiatric hospital admissions within the follow-up period. Secondary outcomes included any compulsory hospital admission (under the UK Mental Health Act (MHA)) and number of days spent in hospital during the follow-up period. These outcome measures were obtained from structured fields within the BRC Case Register. The MHA^23^ is a UK statute law which allows for compulsory admission to hospital for assessment and/or treatment of a mental illness whose nature and/or degree necessitates hospital admission and where a patient does not consent to be voluntarily admitted. Admission under section 2 of the MHA allows for up to 28 days compulsory admission for assessment of mental illness. Admission under section 3 of the MHA allows for up to 6 months compulsory admission for treatment of mental illness. A patient admitted under section 2 may subsequently be placed under section 3 of the MHA. Compulsory hospital admission in this study was defined as admission to a hospital under section 2 or 3 of the MHA.

The number of unique antipsychotic medications prescribed (as a proxy measure of treatment failure) and whether individuals were prescribed clozapine during the follow-up period were also obtained. The number of unique antipsychotics was analysed as a potential mediating factor in determining association of cannabis use with the primary and secondary outcome variables.

The following variables were extracted as covariates for multivariable analyses: age, gender, ethnicity, marital status and diagnosis. All covariate data obtained were those closest to the date of being accepted by an early intervention service. Ethnicity was recorded according to categories defined by the UK Office for National Statistics.^24^ Diagnosis was recorded using the International Classification of Diseases (ICD)-10 classification system, in the following groups: schizophrenia and related disorders (schizophrenia (F20), delusional disorder (F22), schizophrenia-like disorders (F23, F28 and F29)), schizoaffective disorder (F25), mania (F30) or bipolar disorder (F31), psychotic depression (F32.3, F33.3), drug-related psychosis (F1x.5) and other psychotic disorder not otherwise specified. The data were analysed using STATA (V.12) (StataCorp. Stata Statistical Software: Release 12. Coll Station TX StataCorp LP. 2011) using methods described subsequently.

### Follow-up period

Outcome data were collected up to 31 March 2014. All participants were assessed for outcomes within 12 months of the date of being accepted to an early intervention service (2026 person-years). Participants with sufficient follow-up data were also assessed for outcomes within 24 months (n=1738; 3476 person-years), 36 months (n=1461; 4383 person-years), 48 months (n=1185; 4740 person-years) and 60 months (n=926; 4630 person-years). Analyses were performed over discrete periods of follow-up rather than using survival analysis owing to non-proportionality of hazards over time for the clinical outcomes described above and in order to facilitate the exploratory mediation analysis described subsequently.

### Statistical analysis

#### Descriptive statistics

Descriptive statistics for predictor, covariate, mediating and outcome variables were obtained as means and SDs for continuous variables (age and number of inpatient days), means and variances for count variables (number of hospital admissions and number of unique antipsychotic medications), and as frequencies and percentages for all other variables.

#### Associations of cannabis use with demographic factors and clinical outcome

The Mann-Whitney test was used to analyse differences in mean age at presentation (depending on cannabis use) in addition to analysis of age as a categorical variable in regression analyses. Owing to overdispersion (see supplementary material: eTables 1–5), associations with number of hospital admissions and number of unique antipsychotic medications were analysed using multivariable negative binomial regression. Although there was an excess of zero values for number of hospital admissions at 1-year, 2-year and 3-year follow-up, fitting a zero-inflated negative binomial regression model resulted in no meaningful difference compared with standard negative binomial regression (Vuong p>0.05 for all models). The association of cannabis use with compulsory hospital admission was assessed using multivariable binary logistic regression. Association with number of inpatient days was assessed using multiple linear regression. Reference groups for covariates in regression analyses were defined as those with the greatest prevalence within each variable. Where covariate data were not recorded (83 participants with unrecorded marital status), this was included as a predictor variable in regression analyses. No patients were dropped from analyses due to missing covariate data.

#### Mediation of outcomes by antipsychotic treatment failure

**Figure 1:**
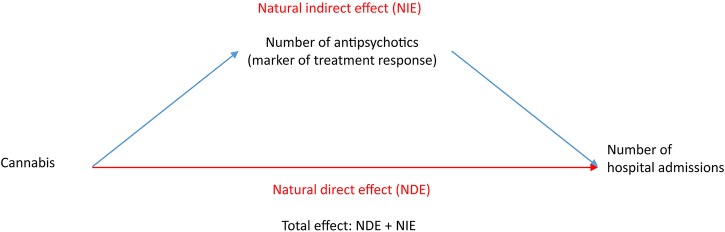
Mediation analysis.

In order to test the potential mediation of the effect of cannabis use on outcome variables by antipsychotic treatment failure, an exploratory mediation analysis was performed using the PARAMED module in STATA.^25^ This is an extension of the Baron and Kenny method^26^ in which a regression model examining the association between the proposed mediator variable and predictor variable is compared with a regression model examining the association between the outcome and the predictor together with the proposed mediator variable. A counterfactual framework which allows for interactions between the exposure and mediator variables is then used to compare the two models to estimate the direct effect of the predictor variable on outcome and the indirect effect of the predictor variable on outcome via the proposed mediator variable.^27^ Comparison of the magnitude of the direct and indirect effect allows for estimation of the proportion of total effect that is mediated. In this study, the number of unique antipsychotics (a proxy measure of treatment failure) was selected as a potentially mediating variable of the effect of cannabis use on outcomes (analysed as a linear variable), with age, gender, diagnosis, ethnicity and marital status as covariates. The results are reported as the natural direct effect of cannabis use on outcomes, the natural indirect effect of cannabis use on outcomes mediated by number of unique antipsychotics, and the estimated total effect representing the combined natural direct and indirect effect (figure 1). The percentage of the total effect mediated by number of unique antipsychotics was estimated for the number of days spent in hospital by dividing the natural indirect effect estimate by the total effect and for the number of admissions to hospital and compulsory hospital admission by dividing the natural logarithm of the natural indirect effect by the natural logarithm of the total effect.

## Results

### Cannabis use among individuals with FEP

Of the total sample, 939 individuals (46.3%) with FEP were found to have a documented history of cannabis use at presentation to early intervention services. Table 1 shows the breakdown of cannabis use by age, gender, ethnicity, marital status and diagnosis. In a multivariable logistic regression analysis (table 1), cannabis use was independently associated with the 16–25-year age group, male gender, single marital status and with a diagnosis of drug-induced psychosis. Cannabis users presented at a younger age than those without documented cannabis use (23.8 vs 24.9 years, Mann-Whitney z=3.84, p<0.001). There was no significant association of cannabis use with ethnicity and cannabis use was less likely among those with psychotic depression or other psychotic disorder not otherwise specified than those with a diagnosis of schizophrenia.

**Table 1:** Multivariable logistic regression analysis of clinical and demographic factors and history of cannabis use at presentation with first episode psychosis (n=2026)

Factor	Number in sample	Percentage with history of cannabis use (%)	Univariate analysis OR (95% CI), p value	*Multivariable analysis OR (95% CI), p value
Age <16 years	19	10.5	0.11 (0.03 to 0.48), p=0.003	0.12 (0.03 to 0.53), p=0.005
Age 16–25 years	1234	51.7	Reference	Reference
Age 26–35 years	747	39.0	0.60 (0.50 to 0.72), p<0.001	0.70 (0.57 to 0.85), p=0.017
Age >35 years	26	30.8	0.42 (0.18 to 0.96), p=0.04	0.48 (0.20 to 1.14), p=0.006
Female	731	30.5	0.35 (0.29 to 0.43), p<0.001	0.39 (0.32 to 0.48), p<0.001
Male	1295	55.3	Reference	Reference
White	616	49.8	1.21 (0.99 to 1.48), p=0.06	1.17 (0.95 to 1.45), p=0.15
Asian	126	38.9	0.78 (0.53 to 1.13), p=0.19	0.84 (0.56 to 1.25), p=0.38
Black	1005	45.1	Reference	Reference
Other	279	46.6	1.06 (0.81 to 1.39), p=0.65	1.13 (0.84 to 1.50), p=0.42
Married/cohabiting	153	28.8	0.41 (0.28 to 0.59), p<0.001	0.56 (0.38 to 0.82), p=0.003
Divorced/separated	63	23.9	0.32 (0.18 to 0.57), p<0.001	0.47 (0.26 to 0.87), p=0.02
Single	1727	49.4	Reference	Reference
Marital status not recorded	83	31.3	0.47 (0.29 to 0.75), p=0.002	0.50 (0.30 to 0.82), p=0.006
Schizophrenia and related	1097	48.4	Reference	Reference
Bipolar disorder	100	52.0	1.15 (0.77 to 1.74, p=0.49)	1.44 (0.93 to 2.22), p=0.10
Psychotic depression	94	30.9	0.48 (0.30 to 0.75, p=0.001)	0.56 (0.35 to 0.90), p=0.02
Schizoaffective disorder	35	34.2	0.56 (0.27 to 1.13, p=0.10)	0.72 (0.35 to 1.51), p=0.39
Drug-induced psychosis	63	79.0	4.10 (0.62 to 0.92, p<0.001)	3.12 (1.64 to 5.88), p<0.001
Other psychotic disorder	637	41.6	0.76 (0.62 to 0.92, p=0.006)	0.79 (0.64 to 0.97), p=0.02

*Multivariable analysis adjusted for all factors presented in table (and no others).

### Hospital admission

**Figure 2:**
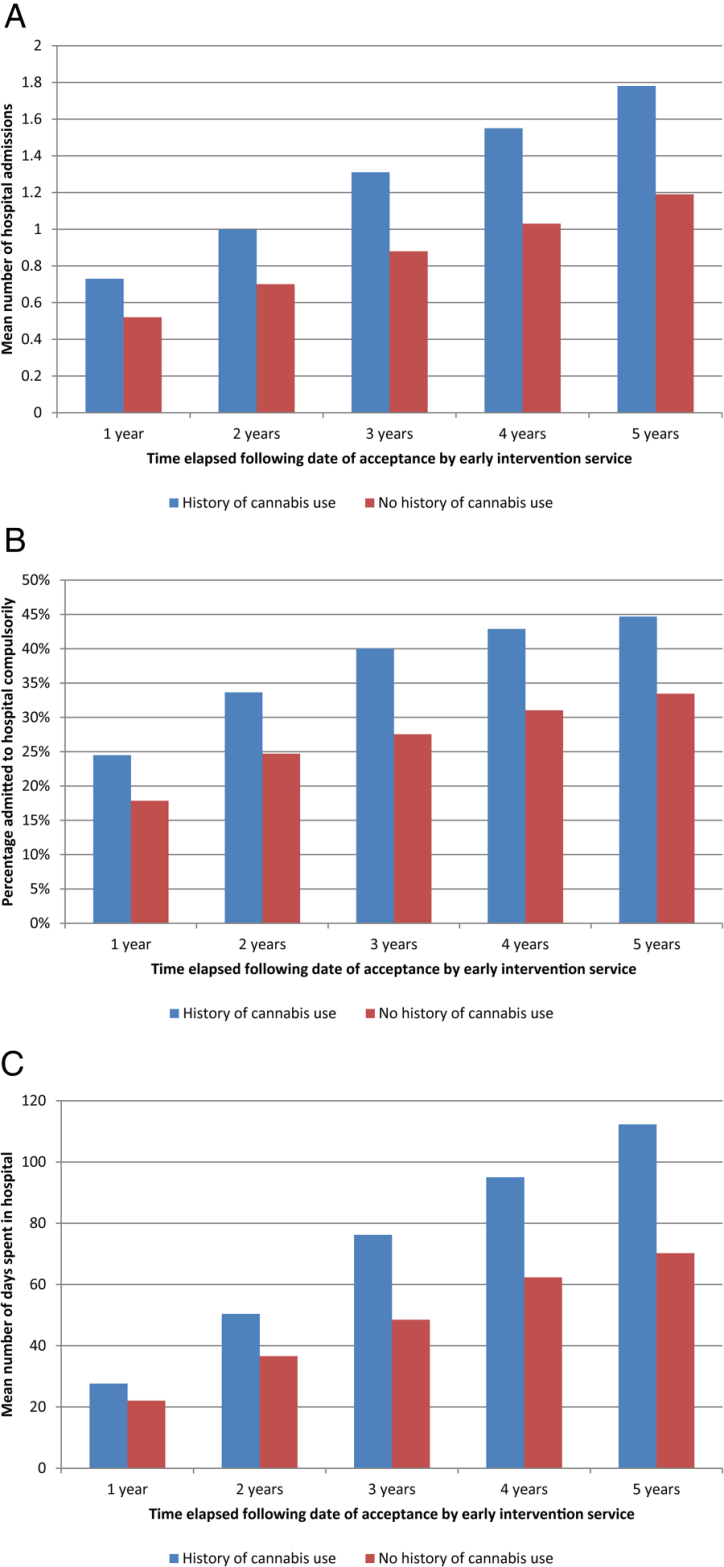
(A) Mean number of hospital admissions among individuals with first episode psychosis with and without documented cannabis use at presentation. (B) Cumulative percentage of patients with first episode psychosis admitted to hospital compulsorily under the UK Mental Act with and without documented cannabis use at presentation. (C) Mean number of days spent in hospital following first episode psychosis depending on history of cannabis use at presentation.

Figure 2A, B illustrate the mean number of hospital admissions and likelihood of compulsory hospital admission (under the UK MHA) up to 5 years following presentation. Corroborated by multivariable regression analyses (table 2), a recorded history of cannabis use was associated with a significant increase in the number of hospital admissions each year after presentation up to year 5, and a significantly increased likelihood of compulsory hospital admission. The data also showed a greater mean number of days spent in hospital, significant from year 2 onwards, following presentation with a history of cannabis use (figure 2C).

**Table 2:** Multivariable analyses of relationship between history of cannabis use at presentation with first episode psychosis and frequency of hospital admissions, likelihood of compulsory hospital admission and mean number of days spent in hospital

Follow-up period	Number in sample	*Number of admissions to hospital Incidence rate ratio (95% CI), p value	†Compulsory hospital admission OR (95% CI), p value	‡Number of days spent in hospital β coefficient (95% CI), p value
1 year	2026	1.37 (1.21 to 1.56), p<0.001	1.33 (1.06 to 1.67), p=0.02	4.1 (−0.6 to 8.7), p=0.09
2 years	1738	1.40 (1.23 to 1.59), p<0.001	1.45 (1.16 to 1.81), p=0.001	9.6 (0.7 to 18.5), p=0.03
3 years	1461	1.48 (1.28 to 1.70), p<0.001	1.65 (1.30 to 2.09), p<0.001	21.6 (8.5 to 34.8), p=0.001
4 years	1185	1.51 (1.29 to 1.76), p<0.001	1.56 (1.20 to 2.02), p=0.001	24.1 (6.1 to 42.0), p=0.009
5 years	926	1.50 (1.25 to 1.80), p<0.001	1.55 (1.16 to 2.08), p=0.003	35.1 (12.1 to 58.1), p=0.003

Results adjusted for age, gender, ethnicity, marital status and psychotic diagnosis.

*Multivariable negative binomial regression.

†Multivariable logistic regression.

‡Multiple linear regression.

### Exploratory mediation analysis

Cannabis use was associated with an increased cumulative likelihood of clozapine (see supplementary material: eTable 4) and number of unique antipsychotics (see supplementary material: eTable 5) prescribed up to 5 years following first presentation. The number of unique antipsychotics prescribed during this period ranged from 0 to 11. While there were no statistically significant differences in clozapine prescription on multivariable logistic regression analysis (see supplementary material: eTable 6), multivariable negative binomial regression (see supplementary material: eTable 7) indicated that a history of cannabis use was associated with an increase in number of unique antipsychotic prescriptions per patient. The exploratory mediation analysis revealed that at 5-year follow-up (table 3) the total effect of cannabis on outcomes was partially mediated by the number of unique antipsychotics prescribed. This was indicated by a significant natural indirect effect of the mediated pathway (figure 1) for each of the three outcomes. The effect of mediation was greatest for the number of days spent in hospital where number of unique antipsychotics (17.9 days, 95% CI 2.4 to 33.4) mediated 51.4% of the total effect (34.8 days, 95% CI 11.6 to 58.1). Outcomes ascertained at follow-up prior to 5 years (see supplementary material: eTable 8) also indicated similar findings with respect to the mediation effect of number of unique antipsychotics on hospital admission outcomes. However, care should be taken in interpreting these findings owing to the possibility of unmeasured confounding and temporal ambiguity between the mediator and outcome variables.

**Table 3:** Mediation analysis investigating association of history of cannabis use at presentation with clinical outcomes mediated by number of unique antipsychotics prescribed at 5-year follow-up (n=926)

Outcomes at 5-year follow-up	Natural direct effect (95% CI, p value)	Natural indirect effect (95% CI, p value)	Total effect (95% CI, p value)	Percentage of total effect mediated by number of unique antipsychotics
Number of admissions to hospital: incidence rate ratio	1.37 (1.12 to 1.68, p=0.002)	1.09 (1.01 to 1.18, p=0.03)	1.50 (1.21 to 1.87, p<0.001)	21.3
Compulsory hospital admission: OR	1.39 (0.68 to 2.83, p=0.37)	1.27 (1.03 to 1.58, p=0.03)	1.76 (0.81 to 3.84, p=0.15)	42.3
Number of days spent in hospital: β coefficient (days)	17.0 (−1.5 to 35.4, p=0.07)	17.9 (2.4 to 33.4, p=0.02)	34.8 (11.6 to 58.1, p=0.003)	51.4

Natural direct effect: cannabis use → outcome.

Natural indirect effect: cannabis use → number of unique antipsychotics → outcome.

Total effect: combined natural direct and natural indirect effect.

## Discussion

We investigated the impact of cannabis use on outcome as indexed by the number of hospital admissions following onset of illness in a large sample of patients with FEP. The analysis captured data for all 2026 residents who received treatment from early intervention services in four London boroughs over an 8-year timeframe and who had been followed up for up to 5 years.

The use of data recorded in electronic health records presented some challenges in conducting the present study. In particular, the ascertainment of cannabis use was dependent on documentation by a healthcare professional in the course of delivering mental healthcare. Despite this, it was possible to identify cannabis use from electronic health records with a high level of precision. The prevalence of a documented history of cannabis use within 1 month of acceptance by early intervention services was 46.3% in the present study. This is consistent with the high levels of lifetime cannabis use reported in other FEP studies (West London 63%^28^; Cambridge 80.3%^29^). However, it is possible that the prevalence identified in our study underestimated cannabis use owing to under-reporting by patients during clinical assessment. The sample demographic was characteristically young and male, converging with demographic characteristics of other FEP cohorts.^4^ ^10^ ^30^ ^31^

Our findings suggest that patients with a history of cannabis use recorded at presentation to an early intervention service were more likely to be admitted to hospital, to require compulsory admission to hospital, and to spend longer in hospital in the 5 years following presentation. We demonstrated an association between cannabis use and the number of different antipsychotics prescribed during the follow-up period (a proxy marker for treatment failure). Finally, the association between cannabis use and the number of unique antipsychotics was found to mediate the increased risk of subsequent hospitalisation, particularly with respect to number of days spent in hospital.

In the present study, it was not possible to establish on the basis of data recorded in electronic health records whether patients were deemed by clinicians to be resistant to a given antipsychotic following a treatment trial at an adequate dose for an adequate duration before they were changed to another. It is also possible that change to a new antipsychotic may have been prompted by admission to hospital due to a relapse. Nevertheless, change to a different antipsychotic, whether as a result of treatment resistance or poor tolerability, suggests a clinical judgement of failure of treatment with the previous antipsychotic. Regardless of whether the change to a new antipsychotic medication occurred in the community or after an admission to hospital, it is likely that any change in antipsychotic represented a failure of treatment, which must have preceded relapse of illness and hospital admission. Together, these results based on clinical decisions documented by clinicians unbiased by awareness of the objectives of the present study, suggest that cannabis use may be associated with increased risk of hospitalisation in psychosis due to an association with antipsychotic treatment failure. There are a number of ways in which cannabis use may have been associated with antipsychotic treatment failure as suggested by the use of multiple different antipsychotics, including a poor response to treatment, poor adherence to treatment and the presence of adverse side effects. Recent studies have linked a poor response to antipsychotic treatment to the presence of a non-dopaminergic pathophysiology in a subgroup of patients with psychotic disorders.^32^ It is possible that increased cannabis use among people with greater number of unique antipsychotics could reflect reduced dopamine synthesis capacity^33^ which could reduce response to dopamine receptor blocking antipsychotic medications. Another possibility is that poor medication adherence among such individuals could have an influence on increased number of unique antipsychotics.^5^ ^12^ It is noteworthy that in our study, cannabis was associated with an increased likelihood of compulsory hospital admission. A previous study suggests that poor medication adherence is associated with compulsory admission and might also explain its association with cannabis use.^34^ While we were not able to tease apart the precise contribution of these various factors to antipsychotic treatment failure, future studies would need to focus on this area, as this may help develop newer strategies for addressing the harmful effects of cannabis.

There are some limitations which should be considered in interpreting the results of this study. The findings presented in this study are based on observational, prospectively recorded clinical data. For this reason, it is not possible to infer any aetiological association between cannabis use and greater risk of hospitalisation or treatment failure. However, it would not be feasible or ethical to conduct a randomised controlled trial to investigate the impact of cannabis use on clinical outcomes. We sought to investigate the potential mediation of relapse (indexed by hospital admission) by treatment failure (indexed by the number of unique antipsychotics prescribed). It is possible that switch to a new antipsychotic may have occurred after hospital admission thereby resulting in reversal of mediator and outcome. However, even in cases where switch to a new antipsychotic may have occurred after hospitalisation, it is extremely unlikely that hospital admission triggered the treatment failure that resulted in a need to change antipsychotic therapy. This implies that even in cases where documentation of a change in antipsychotic occurs after hospital admission, the failure of treatment still occurred prior to admission, and so this may have affected the validity of the exploratory mediation analysis resulting in an underestimate of the effect of number of unique antipsychotics on hospital admission outcomes.

Although we sought to adjust multivariable analyses for potentially confounding factors including age, gender, ethnicity, marital status and diagnosis, there may be other unmeasured confounding genetic and environmental factors (including use of alcohol or other illicit substances) which may have influenced the association of cannabis use with outcomes, as well as differences in positive and negative symptom dimensions which we were unable to measure in our study. Unmeasured confounding may also have affected the results from the exploratory mediation analysis investigating number of unique antipsychotics and the association of cannabis on clinical outcomes. For this reason, it is not possible to conclude that antipsychotic treatment failure is the greatest determinant of poor clinical outcomes in relation to cannabis use and there are likely to be other genetic and environmental factors that could influence the effect of cannabis on clinical outcomes in FEP.

In the present study, we investigated the association of cannabis use documented at presentation with FEP with future clinical outcomes. This was defined as cannabis use in clinical documents recorded within 1 month of presentation to early intervention services. Within the first month of presentation, all participants are likely to have undergone a comprehensive clinical assessment allowing systematic ascertainment of documented cannabis use across the whole cohort at inception. However, this method may have underestimated cannabis use owing to under-reporting by patients during clinical assessment. A further bias may have been introduced by selective documentation of assessing clinicians such that documentation of cannabis use was more likely if it was deemed to be of relevance to a patient's clinical presentation.

It is possible that cannabis use varied during the period of follow-up with some people ceasing to use cannabis and others starting to use it. Previous studies suggest that discontinuation of cannabis is associated with improved clinical outcomes in people with FEP^35^ ^36^ and bipolar disorder.^37^ However, owing to varying level of engagement with mental health services, varying degrees of illness severity and emigration outside the catchment area of clinical services, it was not possible to systematically ascertain ongoing cannabis use in clinical records analysed in this study. It may be that future long-term outcomes were influenced by changes in cannabis use over time. However, if this were the case, it is likely that such variation would have diluted associations with clinical outcomes based on assignment of cannabis use at first presentation to clinical services. It is therefore noteworthy that differences in outcomes based on a history of cannabis use at presentation persisted even at 5-year follow-up. In fact, preliminary analysis of ongoing work in patients with FEP from the same catchment area (n=95) that includes systematic documentation of continuing cannabis use over the follow-up period (by combining clinical records as in the present study with face-to-face research interviews) suggest that 70% of patients with a history of cannabis use at presentation with FEP continued to use cannabis after 3 years, with no new cannabis users who started using following onset of FEP.^38^ Hence, taking into consideration the effect of continuing cannabis use would not have changed the direction of the results reported here, but rather would have demonstrated a stronger adverse effect of cannabis use on outcome in FEP.

Using the cannabis NLP application, it was possible to determine history of cannabis use at presentation with FEP, but it was not possible to determine frequency or amount of cannabis use as this was not systematically recorded in the electronic health record data analysed in this study. Despite this, our findings demonstrated that any cannabis use was significantly associated with poor clinical outcomes, and while the strength of this association may have been greater with increased amount and frequency of cannabis use, such variation is unlikely to have substantially altered the overall association of any cannabis use with poor clinical outcomes that we report here.

These limitations are balanced with the strengths of investigating cannabis use in a large sample of all individuals receiving mental healthcare in early intervention services. Our findings are therefore directly relevant to people who receive care for psychotic disorders in standard clinical practice. The findings presented in this study highlight a clear association between cannabis use and hospitalisation in people with FEP. The fact that over 5 years, cannabis use is associated with 35 additional days spent in hospital has important implications for affected individuals as well healthcare service providers, particularly as almost half of the participants in our study had a history of cannabis use at presentation to early intervention services. This also is the first published study to demonstrate the potential mediation of cannabis use with poorer outcomes by a failure of antipsychotic treatment, albeit with the limitations described previously. Taken together, these findings highlight the importance of ascertaining cannabis use in people receiving care for psychotic disorders and prompt further study to investigate the mechanisms underlying poor clinical outcomes in people who use cannabis and strategies to reduce associated harms.
